# *TP53* Mutations and Phosphatidylinositol 3-Kinase/AKT Pathway Alterations Are Key Determinants of Breast Cancer Outcome Independent of Subtype and Stage

**DOI:** 10.1200/PO-24-00767

**Published:** 2025-05-22

**Authors:** Tibor A. Zwimpfer, Martin Heidinger, Ricardo Coelho, Nadja Stiegeler, Fabienne D. Schwab, Céline Montavon, Ruth S. Eller, Nadia Maggi, Julie M. Loesch, Marcus Vetter, Matteo Lambertini, Walter P. Weber, Christian Kurzeder, Viola Heinzelmann-Schwarz

**Affiliations:** ^1^ Gynecological Cancer Centre, University Hospital Basel and University of Basel, Basel, Switzerland; ^2^ Department of Biomedicine, University of Basel, Basel, Switzerland; ^3^ Breast Centre, University Hospital Basel and University of Basel, Basel, Switzerland; ^4^ Department of Gynecology and Obstetrics, Bethesda Hospital Basel, Basel, Switzerland; ^5^ Medical Oncology, Cantonal Hospital Baselland, Medical University Clinic, Liestal, Switzerland; ^6^ Department of Internal Medicine and Medical Specialties (DIMI), School of Medicine, University of Genova, Genova, Italy; ^7^ Department of Medical Oncology, U.O.C. Clinica di Oncologia Medica, IRCCS Ospedale Policlinico San Martino, Genova, Italy

## Abstract

**PURPOSE:**

Breast cancer (BC) is a heterogeneous disease with genetic alterations influencing prognosis and treatment response. *TP53* mutations (*TP53*muts) are present in approximately 30% of BC, but their prognostic impact remains controversial. In addition, the phosphatidylinositol 3-kinase (PI3K)/Ak strain transforming (AKT) pathway is frequently altered and represents a promising therapeutic target for BC. Understanding the combined prognostic impact of *TP53*mut and PI3K/AKT pathway alterations across BC subtypes remains underexplored.

**METHODS:**

This retrospective cohort study integrated clinical and genomic data from 4,265 patients with BC from the Molecular Taxonomy of Breast Cancer International Consortium (n = 2,509) and the Memorial Sloan Kettering Cancer Center (n = 1,756). Genetic profiling identified *TP53*mut and PI3K/AKT pathway alterations (*AKT1*, *AKT2*, *AKT3*, *PIK3CA*, *PTEN*, *RICTOR*). Survival outcomes were assessed using Kaplan-Meier survival analysis and multivariable Cox proportional hazards models.

**RESULTS:**

In 3,807 patients with available gene alteration status, *TP53*mut was associated with younger age, higher tumor grade, advanced stage, and aggressive subtypes (*P* < .001). *TP53*mut was associated with worse survival independent of subtype, stage, age, and grade (hazard ratio [HR], 1.43 [95% CI, 1.24 to 1.66]; *P* < .0001). The type of *TP53*mut has also been found to be prognostic in BC. PI3K/AKT pathway alterations were more frequent in *TP53*mut tumors and independently associated with worse survival (HR, 1.18 [95% CI, 1.03 to 1.35]; *P* = .0173). The combined presence of *TP53*mut and PI3K/AKT alterations resulted in the worst survival outcomes (HR, 1.61 [95% CI, 1.32 to 1.97]; *P* < .0001).

**CONCLUSION:**

*TP53*mut status is a critical prognostic factor in BC, independent of subtypes and stage, and its adverse impact is amplified by PI3K/AKT pathway alterations. These findings emphasize the integration of genetic profiling into routine clinical practice to refine treatment strategies and identify potential therapeutic targets for this high-risk population.

## INTRODUCTION

Breast cancer (BC) is a highly heterogeneous disease, comprising various molecular subtypes with distinct prognoses and therapeutic responses.^1-6^ The genetic landscape of BC includes genomic alterations in several key genes that drive tumor development and progression.^7-14^ Among them, the *TP53* gene plays a crucial role in cell cycle regulation, DNA repair, and apoptosis.^15-17^ Mutations in *TP53* (*TP53*muts) are prevalent in various cancers, including BC, and are often associated with aggressive tumor characteristics and poor prognosis.^18-20^ Approximately 30% of patients with BC have *TP53*mut.^20,21^ The prognostic role of *TP53*mut in BC remains unclear. While most reports suggest poor clinical outcomes for patients with tumors harboring *TP53*mut, others have reported neutral or even beneficial outcomes.^20^ Despite the high prevalence of *TP53*mut in BC, they are not yet used routinely as predictive markers because their effects on outcomes vary because of different biological responses to treatments like DNA damage and hormone deprivation.^20^

**CONTEXT**

**Key Objective**
To determine the prognostic impact of *TP53* mutations and phosphatidylinositol 3-kinase (PI3K)/Ak strain transforming (AKT) pathway alterations across breast cancer (BC) subtypes and assess their combined effect on survival outcomes.
**Knowledge Generated**
Using data from 4,265 patients with BC, this study demonstrates that *TP53* mutation is an independent predictor of poor survival, irrespective of BC subtype and tumor stage. The presence of PI3K/AKT pathway alterations further exacerbates the adverse prognosis associated with *TP53* mutations. Notably, the type of *TP53* mutation was also found to be prognostic in both early- and advanced-stage BC. Worst survival outcomes were observed in patients with tumors harboring both *TP53* mutations and PI3K/AKT pathway alterations.
**Relevance**
These findings emphasize the importance of integrating genomic profiling into routine clinical practice for risk stratification and therapeutic decision making. Given the emerging role of PI3K/AKT inhibitors, this study highlights the potential of targeted therapies in high-risk patients with *TP53* mutations and PI3K/AKT alterations, informing future precision oncology approaches.

The phosphatidylinositol 3-kinase (PI3K)/Ak strain transforming (AKT) pathway is another pivotal signaling cascade involved in cell proliferation, survival, and metabolism.^22,23^ Alterations in this pathway, such as mutations in *PIK3CA* and *AKT* or loss of *PTEN*, contribute to oncogenic transformation and resistance to therapy.^23-28^ The PI3K/AKT pathway is frequently altered in BC, and the selective PI3K inhibitor alpelisib and the pan-AKT inhibitor capivasertib, both in combination with fulvestrant, and the mammalian target of rapamycin (mTOR) inhibitor everolimus with exemestane have shown superior efficacy over endocrine therapy alone in hormone receptor–positive advanced BC, leading to regulatory approval based on the SOLAR-1, BOLERO-2, and Capitello-291 trials, respectively.^29-32^ However, it is important to distinguish that everolimus specifically targets mTOR, a downstream component of the PI3K/AKT pathway, rather than directly inhibiting PI3K itself.^26^

While both *TP53*mut and PI3K/AKT pathway alterations have been studied individually, their combined impact on BC outcomes, particularly across different BC subtypes, remains underexplored. There is evidence that the PI3K/AKT pathway may be *TP53*-dependent, further highlighting the importance of studying these genetic alterations together.^33,34^ Understanding the interplay between *TP53*mut and alterations in the PI3K/AKT pathway is critical for the development of effective prognostic tools and therapeutic strategies.

This study aimed to investigate the prognostic significance of *TP53*mut and PI3K/AKT pathway alterations in BC leveraging data from two of the largest, well-characterized BC cohorts: the Memorial Sloan Kettering (MSK) Cancer Center^13^ and the Molecular Taxonomy of Breast Cancer International Consortium (METABRIC).^12,14^ By integrating clinical and genomic data, we seek to elucidate how these genetic alterations influence survival outcomes and to identify potential interactions between *TP53* and the PI3K/AKT pathway across distinct BC subtypes.

## METHODS

### Study Design and Population

This study retrospective analysis integrated clinical and genomic data from 4,265 patients with BC in the MSK (n = 1,756)^13^ and METABRIC (n = 2,509)^12,14^ cohorts to investigate the impact of *TP53*mut and PI3K/AKT pathway alterations on clinicopathologic features and overall survival (OS) across BC subtypes and stages.

Patients in the MSK cohort were diagnosed and treated between 2000 and 2015 (follow-up until 2019), and their clinical and genomic data were made publicly available through cBioPortal for Cancer Genomics. For the METABRIC cohort, patient diagnoses occurred between 1995 and 2010 (follow-up until 2016), and this data set was also publicly accessible through cBioPortal after its initial publication in 2012.

### Patient Treatment and Sampling Selection

Patients in the MSK and METABRIC cohorts were treated according to standard care practices at their respective institutions, with treatments including surgery, chemotherapy, radiotherapy, and targeted therapies where appropriate. For this analysis, patients were selected based on the availability of comprehensive clinicopathologic and genomic data.

### Variables

#### TP53mut Status

In the MSK cohort, *TP53*mut was detected using targeted next-generation sequencing (NGS) panels, specifically the MSK-Integrated Mutation Profiling of Actionable Cancer Targets (IMPACT) assay.^13^ The METABRIC cohort used whole-exome sequencing (WES) to identify *TP53*mut.^12,14^ For our analysis, all types of pathogenic *TP53*mut were included. This comprehensive approach was chosen because different mutation types can have varying effects on the function of the p53 protein, potentially influencing tumor behavior and patient outcomes.^35,36^ Given that *TP53*mut can result in loss of function, dominant-negative effects, or gain of function, considering all mutation types provides a holistic view of their prognostic significance.

#### PI3K/AKT Pathway Alterations

The PI3K/AKT pathway alterations analyzed in this study focused on amplifications and mutations in key regulatory genes: *AKT1*, *AKT2*, *AKT3*, *PIK3CA*, *RICTOR*, and *PTEN*. For *AKT1*, *AKT2*, *AKT3*, *PIK3CA*, and *RICTOR*, only whole-gene amplifications were considered. For *PTEN*, all mutation types, including missense, nonsense, frameshift, and splice site mutations, were included. This distinction was made because amplifications typically result in increased gene expression and activity, whereas *PTEN*, being a tumor suppressor, is more commonly inactivated by mutations.^22,23,28,37^ Amplifications in *AKT1*, *AKT2*, *AKT3*, *PIK3CA*, and *RICTOR* lead to hyperactivation of the PI3K/AKT pathway, promoting oncogenesis.^22,23,37^ By contrast, *PTEN* mutations result in loss of function, leading to pathway activation because of the absence of its inhibitory effect on PI3K/AKT signaling.

In the MSK cohort, gene amplifications and mutations were identified using the MSK-IMPACT assay.^13^ Amplifications were defined as ≥six copies. In the METABRIC cohort, WES and single nucleotide polymorphism arrays were used to detect amplifications (≥six copies) and mutations.^12,14^

#### BC Subtypes

Patients were classified into four BC subtypes based on their hormone receptor and human epidermal growth factor receptor 2 (HER2) status, which were comprehensively available in both the MSK and METABRIC cohorts: hormone receptor+/HER2–, hormone receptor+/HER2+, hormone receptor–/HER2+, and triple-negative BC (hormone receptor–/HER2–), following standard clinical practice.^4,5^ Hormone receptor and HER2 status were determined using immunohistochemistry (IHC) and fluorescence in situ hybridization (FISH) in both the MSK and METABRIC cohorts. Hormone receptor status was considered positive in the tumor expression of at least one of either estrogen receptor or progesterone receptor. HER2 status was determined by IHC and confirmed by FISH if IHC results were equivocal.

Additional clinicopathologic variables included the following:

Age at diagnosis: recorded as continuous data and categorized into median and range.

Tumor grade: classified into grades 1, 2, and 3.

Stage at diagnosis: grouped into early stage (I + II) and advanced stage (III + IV).

Menopausal state: categorized as premenopausal or postmenopausal.

OS: time from diagnosis to death or last follow-up, expressed in years.

### Data Sources and Measurement

Clinical data, including age, tumor grade, stage, menopausal state, and survival outcomes, were obtained from electronic medical records and tumor registries within the MSK and METABRIC cohorts. Genomic data were acquired through NGS techniques used by both institutions.^12-14^ Data were accessed through the cBioPortal for Cancer Genomics, where both clinical and genomic data sets were publicly available.

### Statistical Analysis

Descriptive statistics are presented as counts and frequencies for categorical data and as medians (range) for metric or ordinal variables. Comparative analyses between groups were performed using the Kruskal-Wallis rank-sum test for continuous variables and Pearson's chi-squared or Fisher's exact tests for categorical variables. Statistical significance was set at a *P* value of <.05. Kaplan-Meier survival curves were generated, and differences between groups were assessed using the log-rank test. Survival times were censored at 15 years from diagnosis. Multivariable Cox proportional hazards models were used, and interaction analysis was performed. The results of the Cox models were reported as hazard ratios (HRs) with 95% CIs. *P* values from Cox proportional hazards models correspond to Wald and log-rank tests, and a value of <.05 was considered statistically significant. All statistical analyses were conducted using R software (version 4.1.2).

### Ethics Approval and Consent to Participate

Ethical approval and informed consent procedures for the original data collection were obtained from the original cohorts (MSK and METABRIC). The original cohorts (MSK and METABRIC) obtained informed consent from all participants, including consent for the use of their data in future research. This retrospective study used anonymized data under the umbrella of the original consent agreements. All patient data used in the analysis were anonymized and deidentified to protect patient privacy. Data handling and storage complied with applicable data protection regulations, ensuring that no personal identifiers were accessible to the research team.

## RESULTS

### Descriptive Analysis

In this study, we analyzed 4,265 patients with BC from the MSK and METABRIC cohorts and *TP53*mut and PI3K/AKT pathway alteration status was available for 3,807 patients. The MSK cohort was enriched for advanced-stage disease compared with the METABRIC cohort (41% *v* 8.7%; *P* < .001), had a younger median age (52 *v* 61 years), and poorer survival (median OS, 3 *v* 10 years; Data Supplement, Table S1). The frequency of *TP53*mut was balanced between the cohorts (36% *v* 36%; *P* = .8), whereas the MSK cohort had a lower frequency of PI3K/AKT pathway alterations (12% *v* 35%; *P* < .001). In particular, there was a lower frequency of amplifications of all regulatory genes in the MSK cohort.

When we stratified the combined cohorts by *TP53*mut status, significant differences were observed between the *TP53*mut and *TP53*wt groups (Table 1). Patients with *TP53*mut tumors had a younger median age compared with *TP53*wt (54 *v* 58 years; *P* < .001), higher prevalence of grade 3 tumors (82% *v* 44%; *P* < .001), advanced stage at diagnosis (30% *v* 24%; *P* < .001), and a higher proportion of triple-negative BC (30% *v* 3%; *P* < .001). PI3K/AKT pathway alterations were more common in patients with *TP53*mut versus *TP53*wt tumors (28% *v* 22%; *P* < .001). All included regulatory genes except for *AKT3* showed a significant enrichment of alterations in the *TP53*mut group. OS was shorter in patients with *TP53*mut tumors, with a median OS of 5 years (range, 2-11) compared with 7 years (range, 3-13) in the *TP53*wt (*P* < .001).

**TABLE 1.** Clinicopathologic Characteristics and Selected Genomic Features of the Combined MSK and METABRIC Cohorts Stratified by *TP53* Mutation Status

Variable	No.	*TP53*-Mutated (n = 1,378)	*TP53* Wild-Type (n = 2,429)	*P* ^a^
Age at diagnosis, years	3,804			
Median		54	58	<.001
Range		45-64	48-68	
Unknown		0	3	
Grade, No. (%)	3,550			
G1		17 (1.3)	237 (11)	<.001
G2		224 (17)	999 (45)	
G3		1,082 (82)	991 (44)	
Unknown		55	202	
Stage at diagnosis, No. (%)	3,192			
I + II		789 (70)	1,560 (76)	<.001
III + IV		343 (30)	500 (24)	
Unknown		246	369	
Molecular subtypes, No. (%)	3,622			
Hormone receptor+/HER2–		617 (48)	2,092 (90)	<.001
Hormone receptor+/HER2+		140 (11)	130 (5.6)	
Hormone receptor–/HER2+		148 (11)	35 (1.5)	
Triple-negative		391 (30)	69 (3.0)	
Unknown		82	103	
Menopausal state,^b^ No. (%)	3,504			
Post		769 (61)	1,528 (68)	<.001
Pre		492 (39)	715 (32)	
Unknown		117	186	
PI3K/AKT pathway alteration, No. (%)	3,807			
Yes		385 (28)	544 (22)	<.001
No		993 (72)	1,885 (78)	
*AKT1*, No. (%)	3,807			
Amplified		26 (1.9)	12 (0.5)	<.001
Nonamplified		1,352 (98)	2,417 (78)	
*AKT2*, No. (%)	3,807			
Amplified		28 (2)	8 (0.3)	<.001
Nonamplified		1,350 (98)	2,421 (99.7)	
*AKT3*, No. (%)	3,807			
Amplified		148 (11)	364 (15)	<.001
Nonamplified		1,230 (89)	2,065 (85)	
*PIK3CA*, No. (%)	3,807			
Amplified		78 (5.7)	21 (0.9)	<.001
Nonamplified		1,300 (94.3)	2,408 (99.1)	
*RICTOR*, No. (%)	3,807			
Amplified		44 (3.2)	24 (1)	<.001
Nonamplified		1,334 (96.8)	2,405 (99)	
*PTEN*, No. (%)	3,807			
Mutated		133 (9.7)	163 (6.7)	.001
Wild-type		1,245 (90)	2,266 (93)	
Overall survival, years	3,623			
Median		5	7	<.001
Range		2-11	3-13	
Unknown		82	102	
Cohort, No. (%)	3,807			
METABRIC		746 (54)	1,305 (54)	.8
MSK		632 (46)	1,124 (46)	

Abbreviations: AKT, Ak strain transforming; G, grade; HER2, human epidermal growth factor receptor 2; METABRIC, Molecular Taxonomy of Breast Cancer International Consortium; MSK, Memorial Sloan Kettering; PI3K, phosphatidylinositol 3-kinase.

^a^ The *P* values were calculated using the Kruskal-Wallis rank-sum test (medians) or Pearson's chi-squared and Fisher's exact test (categorical data). A *P* < .05 was considered significant.

^b^ In the METABRIC cohort, menopausal state was inferred using a cutoff age of 50 years.

Stratifying by BC subtype, the median age at diagnosis varied, with patients who were hormone receptor+/HER2+ being the youngest (53 years) and patients who were hormone receptor+/HER2– being the oldest (58 years; *P* < .001; Table 2). The highest proportion of grade 3 tumors was detected in the triple-negative subtype (89%; *P* < .001). Advanced stage was more prevalent in hormone receptor+/HER2+ (39%) and hormone receptor–/HER2+ (36%) compared with hormone receptor+/HER2– (25%) and triple-negative subtypes (23%; *P* < .001). *TP53*mut was most prevalent in the triple-negative (85%) and hormone receptor–/HER2+ (81%) subtypes (*P* < .001). The highest rate of PI3K/AKT pathway alterations was found also in the triple-negative subtype (32%; *P* < .001). The shortest median OS was observed in the triple-negative (4 years; range, 2-12) and hormone receptor–/HER2+ (5 years; range, 3-10) subtypes (*P* < .001).

**TABLE 2.** Clinicopathologic Characteristics and Selected Genomic Features of the Combined MSK and METABRIC Cohorts Stratified by Breast Cancer Subtypes

Variable	No.	Hormone Receptor+ HER2– (n = 2,778)	Hormone Receptor+/HER2+ (n = 278)	Hormone Receptor–/HER2+ (n = 192)	Triple-Negative (n = 488)	*P* ^a^
Age at diagnosis, years	3,736					
Median		58	53	52	54	<.001
Range		48-68	44-63	44-59	44-65	
Grade, No. (%)	3,475					
G1		249 (9.7)	5 (1.9)	1 (0.6)	4 (0.8)	<.001
G2		1,061 (41)	55 (22)	26 (14)	49 (10)	
G3		1,252 (49)	195 (76)	153 (85)	422 (89)	
Unknown		20	216	12	13	
Stage at diagnosis, No. (%)	3,209					
I + II		1,808 (75)	151 (61)	101 (64)	307 (77)	<.001
III + IV		596 (25)	96 (39)	56 (36)	94 (23)	
Unknown		374	31	35	87	
Menopausal state,^b^ No. (%)	3,618					
Post		1,840 (69)	150 (56)	105 (56)	295 (62)	<.001
Pre		842 (31)	119 (44)	83 (44)	184 (38)	
Unknown		96	96	4	9	
*TP53* mutation status, No. (%)	3,622					
Mutated		617 (23)	140 (52)	148 (81)	391 (85)	<.001
Wildtype		2,092 (77)	130 (48)	35 (19)	69 (15)	
Unknown		69	8	9	28	
PI3K/AKT pathway alteration, No. (%)	3,622					
Yes		610 (23)	58 (21)	48 (26)	149 (32)	<.001
No		2,099 (77)	212 (79)	135 (74)	311 (68)	
Unknown		69	8	9	28	
Overall survival, years	3,736					
Median		7	6	5	4	<.001
Range		3-13	3-11	3-10	2-12	
Cohort, No. (%)	3,736					
METABRIC		1,413 (51)	113 (41)	134 (70)	320 (66)	<.001
MSK		1,365 (49)	165 (59)	58 (30)	168 (4)	

Abbreviations: AKT, Ak strain transforming; G, grade; HER2, human epidermal growth factor receptor 2; METABRIC, Molecular Taxonomy of Breast Cancer International Consortium; MSK, Memorial Sloan Kettering; PI3K, phosphatidylinositol 3-kinase.

^a^ The *P* values were calculated using the Kruskal-Wallis rank-sum test (medians) or Pearson's chi-squared and Fisher's exact test (categorical data). A *P* < .05 was considered significant.

^b^ In the METABRIC cohort, menopausal state was inferred using a cutoff age of 50 years.

### Impact of *TP53*muts and PI3K/AKT Pathway Alterations on Survival Across Breast Cancer Subtypes

**FIG 1.**
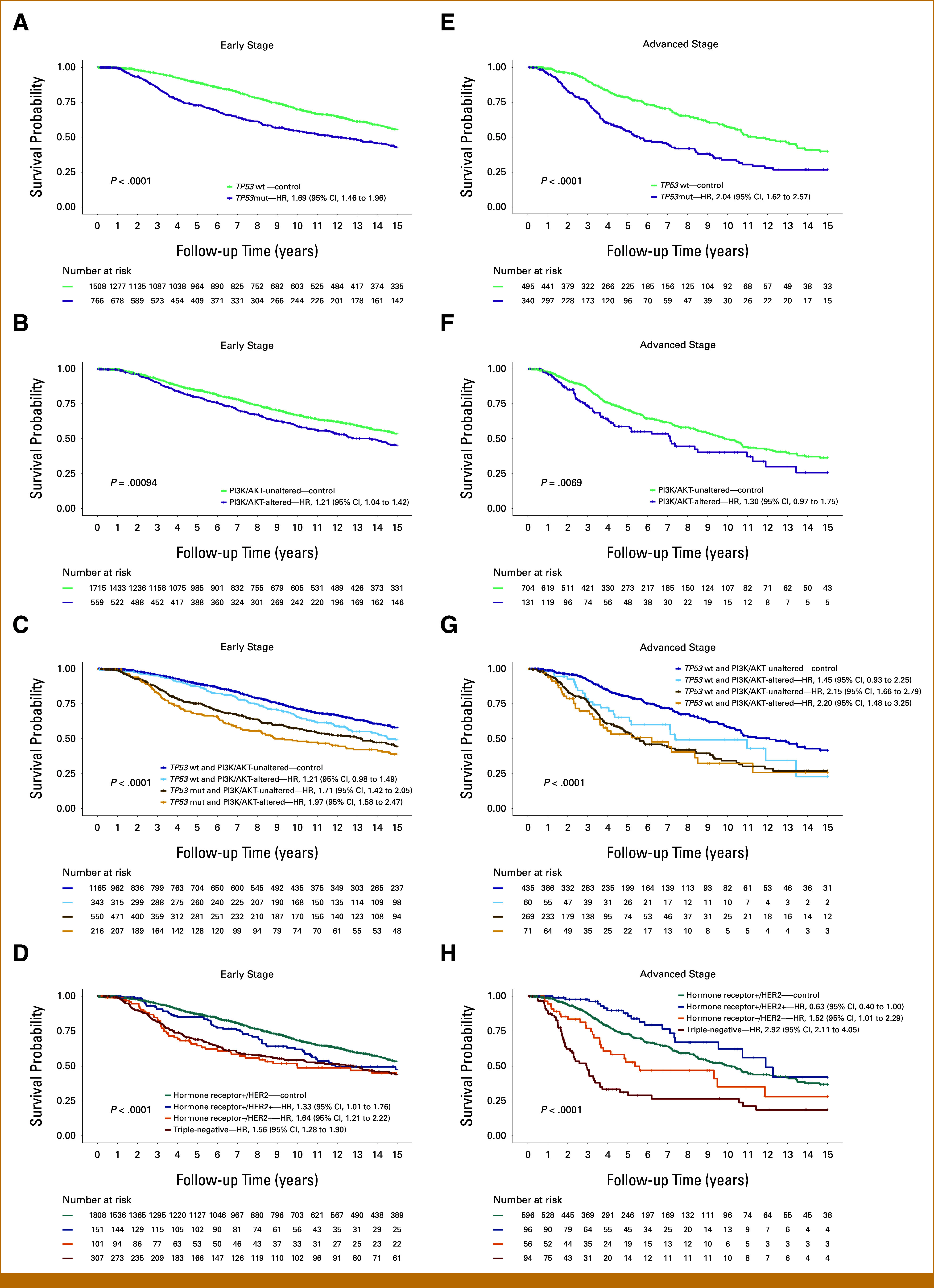
Survival analysis of patients with early- and advanced-stage breast cancer stratified by *TP53* mutation status, PI3K/AKT pathway alteration status, and breast cancer subtypes. The figure presents Kaplan-Meier survival curves analyzing the impact on patient survival in early-stage breast cancer in the left panel by (A) *TP53* mutation status, (B) PI3K/AKT pathway alteration status, (C) combined *TP53* mutation and PI3K/AKT pathway alteration status, and (D) breast cancer subtypes and in advanced-stage breast cancer in the right panel by (E) *TP53* mutation status, (F) PI3K/AKT pathway alteration status, (G) combined *TP53* mutation and PI3K/AKT pathway alteration status, and (H) breast cancer subtypes. AKT, Ak strain transforming; HER2, human epidermal growth factor receptor 2; HR, hazard ratio; mut, mutated; PI3K, phosphatidylinositol 3-kinase; wt, wildtype.

Kaplan-Meier survival curves and unvariable Cox proportional hazards models highlighted the significant associations between genetic alterations, molecular subgroups, and survival outcomes. Patients with *TP53*mut tumors exhibited significantly worse survival compared with *TP53*wt tumors in both early (HR, 1.69 [95% CI, 1.46 to 1.96]; *P* < .0001) and advanced stages (HR, 2.03 [95% CI, 1.62 to 2.57]; *P* < .0001; Figs 1A and 1B; Data Supplement, Fig S1A). The type of *TP53* mutation was also associated with different survival outcomes in both early-stage and advanced-stage BC (Data Supplement, Tables S2 and S3). Alterations in the PI3K/AKT pathway were also associated with worse survival in both early (HR, 1.21 [95% CI, 1.04 to 1.42]; *P* = .0009) and advanced stages (HR, 1.30 [95% CI, 0.97 to 1.75]; *P* = .0069; Figs 1C and 1D; Data Supplement, Fig S1B). Among these, *PTEN* mutations showed the strongest impact on poor survival (HR, 1.35 [95% CI, 1.11 to 1.65]; *P* = .002), whereas *PIK3CA* and *AKT1* amplifications showed a significant interaction with *TP53*mut (*P* interaction = .025 and .034, respectively; Data Supplement, Table S4). The combined presence of *TP53*mut and PI3K/AKT alterations was linked to the poorest survival outcomes in early stages (HR, 1.97 [95% CI, 1.58 to 2.47]; *P* < .0001; Figs 1E and 1F; Data Supplement, Fig S1C).

BC subtypes showed different survival outcomes in early and advanced stages (Figs 1D and 1E; Data Supplement, Fig S1D). In patients with early-stage tumors, the hormone receptor+/HER2– subtype exhibited the best OS, whereas patients with triple-negative and hormone receptor–/HER2+ BC had the poorest survival, with a HR of 1.69 (95% CI, 1.49 to 1.95; *P* < .0001) and 1.76 (95% CI, 1.42 to 2.17; *P* < .0001), respectively. By contrast, in patients with advanced-stage tumors the hormone receptor+/HER2+ subtype showed the best OS (HR, 0.63 [95% CI, 0.40 to 1.00]; *P* < .0001), whereas patients with triple-negative subtype had the poorest survival (HR, 2.92 [95% CI, 2.11 to 4.05]; *P* < .0001).

**FIG 2.**
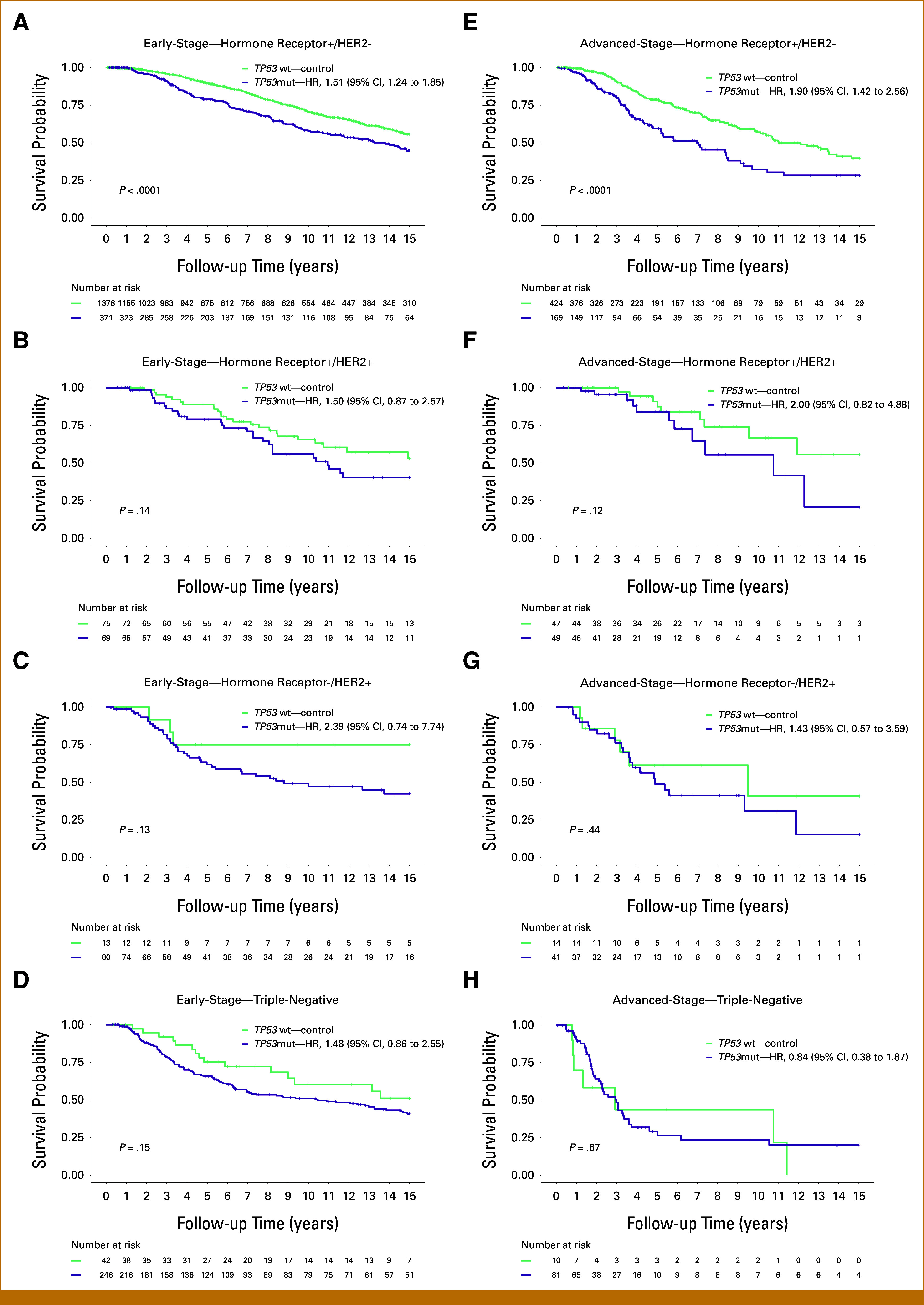
Survival analysis of patients stratified by *TP53* mutation status across the different breast cancer subtypes with early and advanced stages. The figure presents Kaplan-Meier survival curves analyzing the impact on patient survival stratified by *TP53* mutation status in early-stage breast cancer in the left panel for (A) hormone receptor+/HER2–, (B) hormone receptor+/HER2+, (C) hormone receptor–/HER2+, and (D) triple-negative breast cancer subtypes and in advanced-stage breast cancer in the right panel for (E) hormone receptor+/HER2–, (F) hormone receptor+/HER2+, (G) hormone receptor–/HER2+, and (H) triple-negative breast cancer subtypes. HER2, human epidermal growth factor receptor 2; HR, hazard ratio; mut, mutated; wt, wildtype.

**FIG 3.**
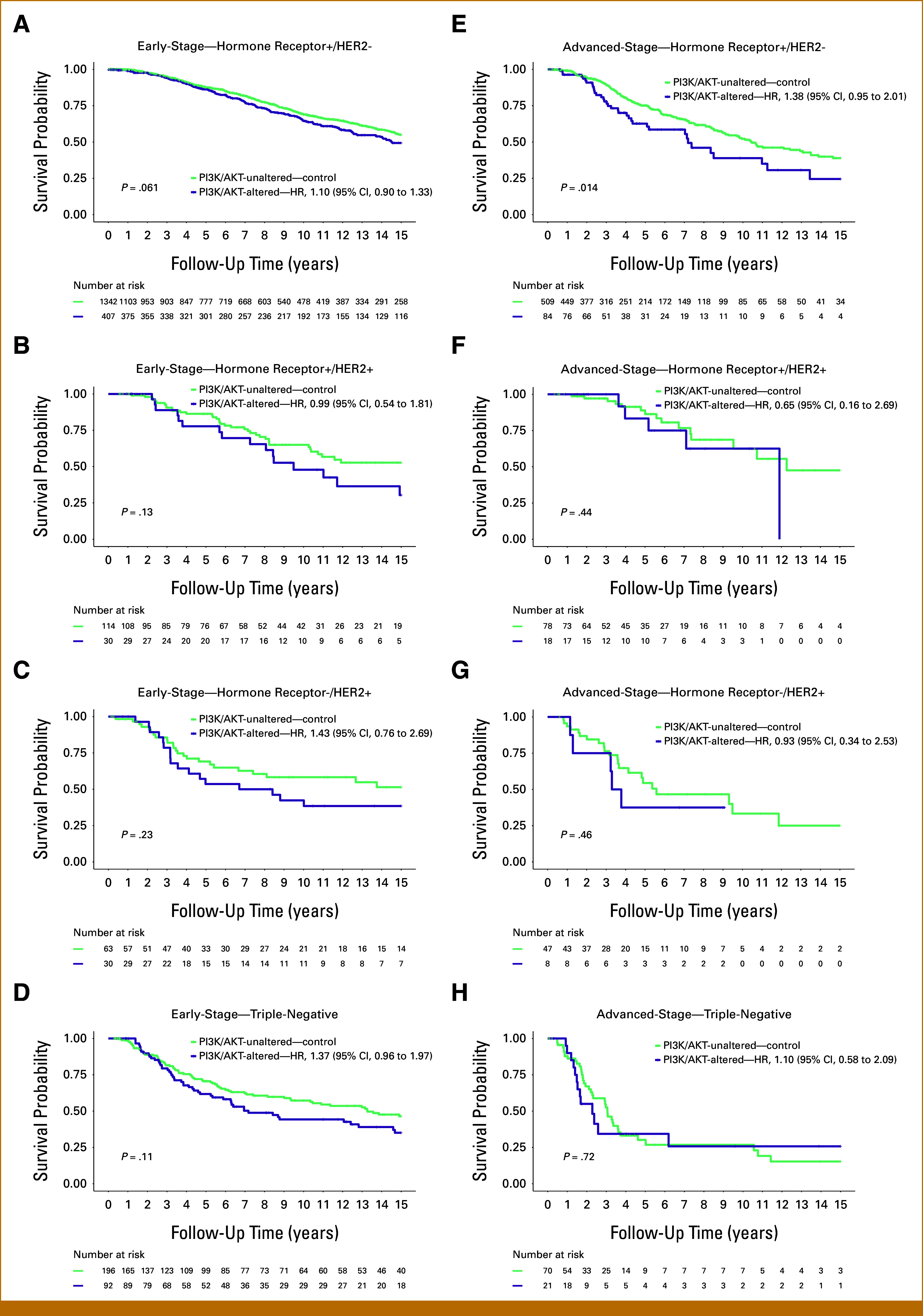
Survival analysis of patients stratified by PI3K/AKT pathway alteration status across the different breast cancer subtypes with early and advanced stages. The figure presents Kaplan-Meier survival curves analyzing the impact on patient survival stratified by PI3K/AKT pathway alteration status in early-stage breast cancer in the left panel for (A) hormone receptor+/HER2–, (B) hormone receptor+/HER2+, (C) hormone receptor−/HER2+, (D) triple-negative breast cancer subtypes and in advanced-stage breast cancer in the right panel for (E) hormone receptor+/HER2−, (F) hormone receptor+/HER2+, (G) hormone receptor−/HER2+, and (H) triple-negative breast cancer subtypes. AKT, Ak strain transforming; HER2, human epidermal growth factor receptor 2; HR, hazard ratio; PI3K, phosphatidylinositol 3-kinase.

BC subtype–specific subanalyses showed a differential impact of *TP53*mut (Figs 2A-2H; Data Supplement, Figs S2A-S2D) and PI3K/AKT pathway alterations (Figs 3A-3H; Data Supplement, Figs S3A-S3D) on survival. In particular, patients with *TP53*mut tumors showed a significant survival disadvantage in the hormone receptor+/HER2– BC subtype for both early (HR, 1.51 [95% CI, 1.24 to 1.85]; *P* < .0001) and advanced stages (HR, 1.90 [95% CI, 1.42 to 1.56]; *P* < .0001). Patients with tumors with alterations in the PI3K/AKT pathway showed in particular a survival disadvantage in the advanced-stage hormone receptor+/HER2– BC subtype (HR, 1.38 [95% CI, 0.95 to 1.38]; *P* = .014; Fig 3E).

### TP53mut and PI3K/AKT Alterations Independently and Synergistically Negatively Affect Survival

**FIG 4.**
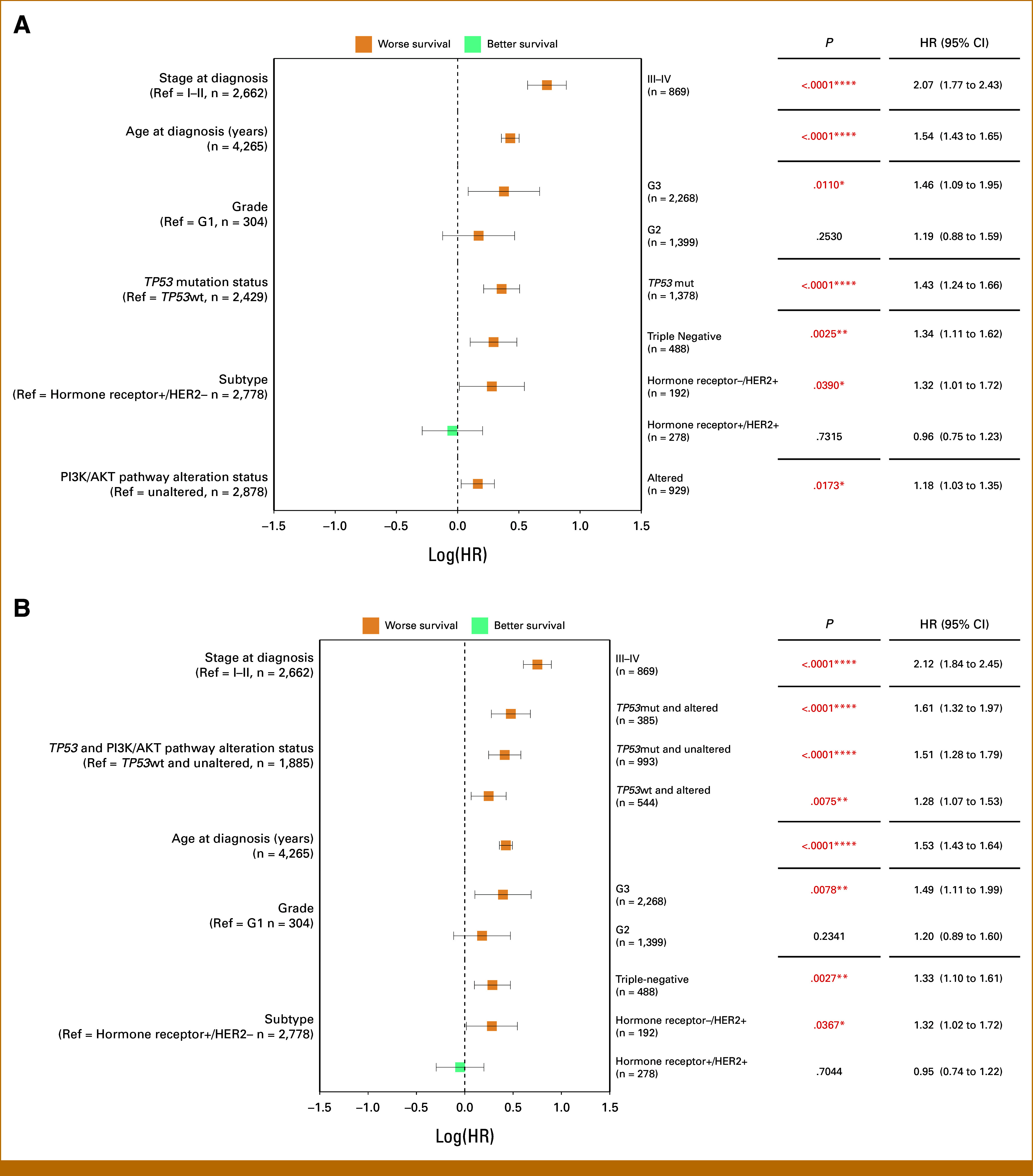
Impact of *TP53* mutations and PI3K/AKT alterations on survival in patients with breast cancer: Independent and combined effects. (A) multivariable Cox proportional hazards model for OS comparing *TP53*-mutated and wildtype and PI3K/AKT pathway–altered and PI3K/AKT pathway–unaltered tumors and including typical clinicopathologic predictive features. (B) multivariable Cox proportional hazards model of the interaction term *TP53* mutation and PI3K/AKT pathway alteration status and clinicopathologic predictive features on OS. The plots depict worse survival (orange) and better survival (blue) indicators for each clinical factor, with *P* values indicating the statistical significance of each association. *P* values < .05 are colored red (*P < .05, **P < .01, ***P < .001, ****P < .0001). The multivariable Cox proportional hazards models were stratified by the cohorts. AKT, Ak strain transforming; G1, grade 1; G2, grade 2; G3, grade 3; HER2, human epidermal growth factor receptor 2; HR, hazard ratio; mut, mutated; OS, overall survival; PI3K, phosphatidylinositol 3-kinase; Ref, reference; wt, wildtype.

A multivariable Cox proportional hazards model was used to adjust for potential confounders and to assess the independent effects of *TP53*mut and PI3K/AKT pathway alterations on OS (Fig 4A). Both *TP53*mut and PI3K/AKT pathway alterations were significantly associated with worse survival outcomes (HR, 1.43 and 1.18, 95% CI 1.24 to 1.66 and 1.03 to 1.35; *P* < .0001 and .0173). The interaction between *TP53*mut and PI3K/AKT pathway alterations was also significant (*P*-interaction = .047; Data Supplement, Table S5). In addition, advanced stage at diagnosis, grade 3 tumors, and the triple-negative subtype were all independently associated with worse survival.

A multivariable Cox regression model was also performed incorporating the interaction term for combined *TP53*mut and PI3K/AKT pathway alteration status based on their significant interaction (Fig 4B). The presence of both, *TP53*mut and altered PI3K/AKT pathway, status also demonstrated a substantial negative impact on survival (HR, 1.61 [95% CI, 1.32 to 1.97]; *P* < .0001). Patients with *TP53*mut tumors without PI3K/AKT alterations and those with *TP53*wt but with PI3K/AKT alterations were significantly associated with poorer survival outcomes (HR, 1.51 [95% CI, 1.28 to 1.79]; *P* < .0001 and HR, 1.28 [95% CI, 1.07 to 1.53]; *P* = .0075).

## DISCUSSION

To our knowledge, this study is the first study to comprehensively evaluate the prognostic significance of *TP53*mut and PI3K/AKT pathway alterations across BC subtypes using data from the MSK^13^ and METABRIC cohorts.^12,14^ Our findings show that both *TP53*mut and PI3K/AKT alterations significantly worsen survival, independent of the BC subtype and other prognostic clinicopathologic features.

*TP53*mut emerged as a key determinant of BC outcomes, retaining a significant association with poorer survival even after adjusting for age, stage, and subtype. *TP53*mut was also associated with more aggressive tumor characteristics, including younger age at diagnosis, higher tumor grade, and prevalence in hormone receptor–/HER2+ and triple-negative BC. These findings align with previous studies associating *TP53*mut with more aggressive tumor behavior and poorer prognosis in BC.^20,38^ Notably, *TP53*mut type was also prognostic in both early- and advanced-stage BC, underscoring *TP53*mut heterogeneity. Different *TP53*mut types may diverge in their functional impact, influencing tumor progression and therapy response.^20,39^

Our study highlights the compounding effect of PI3K/AKT pathway alterations on BC prognosis. These alterations were more frequent in *TP53*mut tumors and independently predicted worse survival. The combined presence of *TP53*mut and PI3K/AKT pathway alterations was associated with the poorest outcomes, suggesting a synergistic effect that exacerbates tumor aggressiveness and therapeutic resistance. Given that *TP53* and PI3K/AKT pathway alterations have been implicated in immune evasion,^40-43^ future research integrating genomic and immune profiling could clarify how these alterations shape the tumor microenvironment and influence immunotherapy responses.

BC subtype analysis provided further insight into the heterogeneous nature of BC. While *TP53*mut and PI3K/AKT pathway alterations worsened survival in all subtypes, their impact varied. *TP53*mut had a greater effect in hormone receptor+/HER2– and hormone receptor+/HER2+ subtypes, whereas its influence was less pronounced in triple-negative and hormone receptor–/HER2+ subtypes, where *TP53*mut is already predominant. Conversely, PI3K/AKT pathway alterations were particularly associated with the poor outcome in HER2+ BC subtypes, underscoring the importance of considering both genetic and hormone receptor/HER2-based subtype context in prognosis and treatment planning.

Patients with tumors with *TP53*mut and PI3K/AKT pathway alterations may benefit from more aggressive surveillance and tailored therapies including PI3K and AKT inhibitors regardless of the BC subtype and stage. Recently, the CAPItello-291 trial^32^ demonstrated positive results for the pan-AKT inhibitor capivasertib in hormone receptor+ BC. In addition, several PI3K/AKT pathway inhibitors are under clinical investigation, reflecting continuous advancements in this area.^44^ Future studies should further evaluate the therapeutic potential of PI3K and AKT inhibitors, particularly in high-risk patients with *TP53*mut and PI3K/AKT alterations. The interaction between *TP53*mut and PI3K/AKT pathway alterations suggests new avenues for combination therapies, such as pairing PI3K/AKT inhibitors with treatments specifically targeting *TP53*mut tumors.

It is important to recognize that treatment paradigms for BC have evolved significantly in recent years.^45-48^ The introduction of new therapies, such as HER2-targeted therapies, CDK4/6 inhibitors, and PARP inhibitors, has improved outcomes, not only for HER2+ and hormone receptor+ but also for triple-negative subtypes.^49-54^ However, given the retrospective nature of the MSK and METABRIC data sets, our analysis may not fully capture the impact of these newer treatments on survival outcomes. Despite this limitation, our study provides important insights into the independent and synergistic effects of *TP53*mut and PI3K/AKT pathway alterations on survival across different BC subtypes, supporting genomic-guided treatment de-escalation or escalation strategies in BC, in particular, in early-stage hormone receptor+ subtype. Future studies should incorporate detailed treatment data to better understand how new targeted therapies interact with genetic alterations, ultimately enabling more personalized and effective treatment approaches for high-risk patients.

This study has several strengths. First, it leverages a large, well-characterized data set from two cohorts, MSK^13^ and METABRIC,^12,14^ allowing for long-term follow-up and increasing the generalizability of findings. Second, the integration of clinical and genomic data enabled a detailed investigation of *TP53*mut, PI3K/AKT pathway alterations, and their interaction with BC subtypes. Third, the use of multivariable Cox proportional hazards models accounted for potential confounders such as age, tumor grade, stage, and BC subtype, enhancing the robustness of survival analyses.^55,56^

However, there are also limitations, primarily because of its retrospective nature, which is subject to inherent biases related to data collection and patient selection.^57,58^ Detailed systemic treatment information, such as the use of new targeted therapies that became available during the study's follow-up period, is lacking. This is a potential confounder, particularly for subtypes like HER2+, where treatment advances have been substantial.^50,53,54,59^ While we accounted for some of these variables by including stage, age, and BC subtype in the multivariable analyses, these variables serve only as proxies for treatment. In addition, differences in treatment regimens between cohorts might have influenced survival outcomes. To minimize this bias, we stratified the analyses by cohort as performed previously.^58,60,61^ We also addressed potential survival bias introduced by deaths potentially unrelated to BC by right censoring at 15 years after diagnosis.

In conclusion, this study highlights the critical prognostic role of *TP53*mut and PI3K/AKT pathway alterations in BC, independent of traditional clinicopathologic factors and BC subtypes. While new therapies have significantly improved survival outcomes, our findings suggest that these genetic alterations remain important determinants of poor prognosis, highlighting the potential benefit of escalation or de-escalation strategies in early-stage BC.

## Data Availability

A data sharing statement provided by the authors is available with this article at DOI https://doi.org/10.1200/PO-24-00767. The data presented in this study are available on request from the corresponding author. Publicly available data sets were analyzed in this study. These data can be found at https://www.cbioportal.org/.
